# Construction of a personality trait prediction model based on color perception preference and personalized cognitive intervention pathways in art education

**DOI:** 10.3389/fpsyg.2026.1876102

**Published:** 2026-08-24

**Authors:** Songhui Yang, Zhongnuo Sun, Ailong Jin, Xiangyang Zhao, Yanchun Xie, Mingyue Li

**Affiliations:** 1 School of Education and Art, Dezhou Vocational College of Science and Technology, Dezhou, Shandong, China; 2 School of Information Engineering, Dezhou Vocational College of Science and Technology, Dezhou, Shandong, China; 3 School of Art and Media, Shenyang Institute of Technology, Shenyang, Liaoning, China; 4 Department of Teaching and Scientific Research, Dezhou Vocational College of Science and Technology, Dezhou, Shandong, China

**Keywords:** art education, attention mechanism, color perception preference, deep learning, personality prediction, personalized intervention

## Abstract

Color perception preference encodes traceable signatures of personality, yet existing approaches to art education seldom translate this link into computationally grounded, individualized instruction. We develop an integrated framework that spans chromatic feature representation, deep-learning-based trait inference, and closed-loop pedagogical intervention. A multidimensional feature system was constructed across HSV and CIE L*a*b* color spaces, combining dominant color centroids, distribution entropy, perceptual contrast, harmony quantification, and an affective coordinate mapping. We propose CA-PPNet, a multi-branch convolutional architecture coupled with multi-head self-attention and five parallel regression heads that jointly predict the Big Five trait dimensions. Trained on 1,247 paired chromatic-personality records, CA-PPNet attained a macro F1 of 0.798 on tertile-binned trait classes and a mean Pearson *r* of 0.731 on the continuous scores—a dual-track evaluation adopted because trait inference straddles rank ordering and magnitude estimation. Ablations confirmed the contributions of multi-branch fusion, attention, and multi-task learning. Predicted trait profiles were then mapped to six trait-anchored intervention pathways. A 12-week experiment with 216 undergraduates, evaluated with a 30-item color cognition test (score range 0–30, four sub-scales: hue identification, contrast judgement, harmony assessment, and affective mapping), yielded a normalized cognitive gain of 0.642 in the personalized arm against 0.418 in a uniform-curriculum control (between-group Cohen’s *d* = 0.91). Prediction error correlated negatively with cognitive gain (*r* = −0.412); because the design cannot exclude third variables such as general ability, we advance this as a hypothesis that intervention efficacy is bounded by trait-inference fidelity rather than as an established mechanism. Because the entire theoretical foundation is culturally situated—color-emotion mappings, personality norms, and the sample itself are all rooted in a Chinese tertiary context—we treat cross-cultural transfer as an acknowledged limitation as well as a future direction. The findings suggest that chromatic preference, when richly represented and modeled with attention-based architectures, can support defensible personalization in studio art instruction and invite fusion with physiological signals and cross-cultural replication.

## 1 Introduction

Color, the most immediate visual stimulus encountered by the human perceptual system, carries information that reaches far beyond aesthetic surface. Long before psychometric instruments were formalized, painters and educators suspected that an individual’s gravitation toward certain hues betrayed something about temperament, mood, and cognitive style (Palmer et al., 2013). Empirical work over the past three decades has gradually substantiated this intuition. Large cross-linguistic studies now report reliable color–emotion regularities with universal cores that are further shaped by linguistic and geographic proximity (Jonauskaite et al., 2020), and machine-learning analyses of individual differences confirm that chromatic preferences carry idiosyncratic yet decodable structure (Jonauskaite et al., 2019b). Meta-analytic and reviewing work on aesthetic engagement places the typical association between chromatic preference and Big Five domains in the modest range of *r* ≈ 0.20–0.35 (Silvia and Beaty, 2020; Specker et al., 2020, 2022), with occasional excursions upward when composite chromatic dimensions replace single-hue predictors. The translation of these correlations into operational tools for art education, however, remains uneven, and this is the gap that motivates the present study.

The application of color psychology in fine arts pedagogy has matured along two largely parallel tracks. One track, rooted in studio practice, treats color as an expressive medium whose emotional resonance is taught through canonical exercises—Itten’s contrasts, Albers’ interaction studies, and their derivatives (Wang and Chen, 2021). The other, situated in educational psychology, examines how learners’ chromatic responses index attentional bias, openness to experience, or affective regulation (Elliot, 2015). Both traditions acknowledge the learner’s interiority, yet they rarely speak to each other in computational terms. The consequence is well documented: instructors often rely on tacit judgement when adapting color exercises to individual students, a practice that resists scaling and verification (Hickman and Brens, 2021).

International research on color-preference measurement has progressed from forced-choice paradigms toward continuous, multi-dimensional assessments. Palmer and Schloss’s ecological valence theory reframed preference as a learned association with object affordances (Schloss and Palmer, 2017; Palmer and Schloss, 2010), while later work integrated eye-tracking and galvanic skin response to capture pre-conscious reactions (Jonauskaite T. et al., 2019). Personality prediction modeling, in turn, has been transformed by deep learning: convolutional and transformer-based architectures now infer trait scores from text, voice, image, and smartphone-behaviour inputs with accuracies that questionnaire-based approaches could not previously approach (Kaushal and Patwardhan, 2018; Stachl et al., 2020; Mehta et al., 2020). Domestic studies in China have contributed culturally sensitive color lexicons and explored their alignment with Confucian-inflected personality dimensions, although sample sizes and modeling depth remain modest (Lee and Kim, 2021; Zhao et al., 2021). Personalized intervention in art education, meanwhile, has been investigated mainly through small-scale pedagogical experiments, with promising but localized results (Park and Lee, 2021).

Three problems persist when these threads are examined together. First, cross-disciplinary integration remains shallow; color science, personality psychology, and pedagogical design are typically combined in narrative reviews rather than in unified computational frameworks (Liu et al., 2021). Prior integrated attempts have either stalled at the level of aesthetic prediction from images without personality outcomes (Bianco et al., 2021), or, in the opposite direction, inferred traits from smartphone behaviour without engaging chromatic content at all (Stachl et al., 2020). Second, predictive accuracy in existing color-based models suffers because chromatic stimuli are reduced to RGB or HSV summaries, discarding the relational structure that observers actually attend to; the wider aesthetics-prediction literature has flagged this reduction as a limiting factor for image-derived inferences of any kind (Bianco et al., 2021; Machajdik and Hanbury, 2019; Lu et al., 2018). Third, intervention pathways tend to be open-loop: a diagnostic profile is generated, instructional adjustments are recommended, but feedback from the learner’s subsequent perceptual behaviour seldom re-enters the system to refine subsequent guidance (Mitchell and Sutherland, 2020). These three deficiencies are not independent; they reinforce one another, and addressing any single one in isolation yields limited returns.

Constructing a personality-prediction model grounded in color perception preference therefore carries both practical and theoretical weight. On the practical side, such a model promises art instructors a defensible, data-informed basis for tailoring exercises, palette suggestions, and critique strategies to individual learners. Theoretically, it offers a testable bridge between low-level perceptual data and high-level dispositional constructs, contributing to debates about whether trait inference can be grounded in perception rather than self-report alone (Rentfrow and Gosling, 2020).

Against this backdrop, the present work advances three contributions and distinguishes them explicitly from prior work. First, we design a multimodal chromatic feature extraction pipeline that jointly preserves chromatic statistics, distributional descriptors, and affective coordinates—the last of these missing from the multi-branch aesthetics predictors reviewed by Mehta et al. (2020) and from the single-space feature systems used in earlier image-based personality models. Second, we develop CA-PPNet, whose combination of semantic-branch tokenisation, multi-head self-attention over branch outputs, and homoscedastic-uncertainty-weighted multi-task heads goes beyond the monolithic CNNs and text-only transformers surveyed by the same authors. Third, we propose a closed-loop cognitive intervention pathway in which predicted trait profiles inform pedagogical adjustments, learner responses are re-encoded through the same multimodal pipeline, and the loop iterates—an arrangement intended to render personalization observable, revisable, and accountable within the studio classroom. These contributions are organised around two distinct research strands with different epistemological statuses. RQ1 is exploratory in spirit and asks whether attention-based chromatic features can predict OCEAN trait scores at levels approaching the theoretical ceiling implied by the reliability of self-report instruments. RQ2 is confirmatory and asks whether trait-anchored routing of learners produces higher normalised cognitive gain than a uniform curriculum over a 12-week window. The hypothesised confirmatory direction for RQ2 is a positive between-arm difference in Hake’s g.

Positioned against this landscape, the paper’s fit with the concerns of Frontiers in Psychology is deliberately narrow. What it offers is a fully specified and reproducible computational pipeline running from perceptual stimulus to instructional decision, together with an evaluation protocol whose stratification, uncertainty quantification, and residual bias audit are reported in full. What it does not offer is a validated psychometric instrument, a culturally transportable model, or a causal warrant for the personalisation effect; each of these is a boundary marked explicitly in Section 5. The contribution is accordingly mathematical and computational in character—a representation, an architecture, an optimisation scheme, and a measurement protocol—and the empirical findings should be read as a first, geographically bounded demonstration rather than as a generalisable result.

## 2 Theoretical foundations of the association between color perception preference and personality traits

### 2.1 Psychological mechanisms of color perception

Color perception begins at the retina, where three classes of cone photoreceptors—long-, medium-, and short-wavelength sensitive—initiate opponent-process channels that the lateral geniculate nucleus and primary visual cortex subsequently elaborate into hue, lightness, and saturation signals (Conway, 2018). Downstream, area V4 and the inferior temporal cortex bind these chromatic dimensions to object identity (Conway, 2018). Chromatic signals then engage the amygdala and orbitofrontal cortex through the same affective pathways that support object and scene valuation more broadly; those pathways are not specific to color, so the emotional charge a hue acquires must be read as one instance of general affective processing rather than as a dedicated color-emotion channel (Jonauskaite et al., 2020; Schloss and Palmer, 2011). This dual arrangement—a perceptual pipeline and a shared affective conduit—helps explain why two viewers with identical retinal input can report markedly different feelings toward the same stimulus.

The three psychophysical attributes of color exert distinguishable effects on emotion and cognition. Hue drives categorical affective associations—warm hues with arousal and approach, cool hues with calm and withdrawal—while lightness modulates perceived weight and openness, and saturation governs the intensity of any emotional response evoked (Jonauskaite et al., 2020). Valdez and Mehrabian’s classical study reported, for pleasure, arousal, and dominance separately, a set of regression equations in which hue angle (in degrees), brightness, and saturation combined additively with dimension-specific weights and interaction terms (Valdez and Mehrabian, 1994). A general parameterisation inspired by their formulation can be written for the arousal dimension as Equation (1):

$$A=\alpha_{1}H+\alpha_{2}L+\alpha_{3}S+\varepsilon$$ (1)

where H, L, and S denote hue angle, lightness, and saturation respectively, *α*₁, α₂, and α₃ are empirically estimated weights, and *ε* captures individual variance (Valdez and Mehrabian, 1994). To underline that this is a general form rather than a re-statement of the original regression, we retain the pleasantness dimension in its simplified illustrative form as Equation (2):

P=0.69L+0.22S (2)

recognising that Valdez and Mehrabian’s own equations included brightness, saturation, and interaction terms with dimension-specific weights that differ across pleasure, arousal, and dominance (Valdez and Mehrabian, 1994). Recent empirical work further shows that the relationship between perceived lightness and psychological functions is substantially more complex than any fixed linear weighting can capture (Bortolotti et al., 2022), so the linear expressions above are used only to motivate the feature engineering that follows, not as production predictors. To accommodate cross-individual deviation, we adopt a normalized preference index as Equation (3):

$$z_{i}=\frac{r_{i}-\bar{r}}{\sigma_{r}}$$ (3)

where rᵢ is the rated preference of participant i for a given chromatic stimulus and r̄, σᵣ are the sample mean and standard deviation. This *z*-score formulation is standard in psychometric color research and allows preference scores from heterogeneous populations to be compared on a common scale.

Several theoretical frameworks have organised these regularities into measurement instruments. The Lüscher Color Test, developed by Max Lüscher in the 1940s and made widely available in English translation later (Lüscher, 1969), infers personality from ranked selections among eight standardised swatches, treating choice order as a projective trace of unconscious need states; Pazda and Greitemeyer offer a critical review of the method’s methodological standing (Pazda and Greitemeyer, 2019). The Color Preference Scale, by contrast, treats preference as a continuous trait variable amenable to factor analysis, while ecological valence theory locates preference in the cumulative affective history a viewer has accrued with objects of that color (Schloss et al., 2018). Each framework rests on different assumptions about whether color responses are universal, learned, or idiosyncratic—a tension we find generative rather than resolvable. Table 1 summarises the four most influential frameworks along axes that matter for the present modeling effort.

**Table 1:** Comparison of classical color psychology theoretical frameworks across measurement paradigm, dimensional structure, and applicability to personality inference.

Framework	Measurement paradigm	Core dimensions	Inference target
Lüscher color test	Forced-choice ranking of 8 swatches	Need states (4 primary, 4 auxiliary)	Unconscious motivational profile
Color preference scale	Likert-scale ratings	Hue, lightness, saturation preference	Continuous trait estimates
Ecological valence theory	Object-color association ratings	Affective valence per hue	Learned preference history
Hering opponent-process model	Psychophysical matching	Red–green, blue–yellow, black–white	Perceptual processing style

The frameworks in Table 1 also reveal why cross-cultural variation cannot be treated as noise. Red, for instance, evokes celebratory associations in much of East Asia yet warning or danger in many Western contexts, and such divergences propagate into personality inferences whenever measurement instruments are transplanted without recalibration (Yu et al., 2018). Individual differences add a further layer: variations in macular pigment density, prior aesthetic training, and even mood at the moment of testing reshape preference responses in ways that any predictive model worth building must accommodate rather than average away.

### 2.2 The Big Five personality trait theory and measurement models

The Big Five, often abbreviated as OCEAN, organises adult personality along five orthogonal dimensions—Openness, Conscientiousness, Extraversion, Agreeableness, and Neuroticism—each treated as a continuous quantitative variable rather than a discrete category (Soto and John, 2017). Openness captures intellectual curiosity, aesthetic sensitivity, and tolerance for ambiguity; Conscientiousness reflects self-discipline, achievement striving, and orderliness; Extraversion concerns sociability, positive affect, and energetic engagement with the external world; Agreeableness indexes cooperative and prosocial tendencies; and Neuroticism describes susceptibility to negative affect and emotional instability (Donnellan et al., 2020). The lexical and factor-analytic traditions from which these dimensions emerged do converge across many languages, yet the picture is not one of uniform stability. De Raad et al. reported that only three of the five factors replicated fully across fourteen trait taxonomies (De Raad et al., 2010), and Gurven et al. (2013) found considerably weaker five-factor structure among forager-farmers in the Bolivian Amazon than in industrialised samples. Because the present study was conducted in China, we relied on a Chinese-language BFI-2 whose psychometric properties in Chinese tertiary samples have been documented by Rammstedt et al. (2020), and we treat the cross-cultural transportability of trait interpretations as an empirical question rather than a settled matter.

Operationalising the Big Five requires reliable instruments. The NEO-PI-R, comprising 240 items grouped into 30 narrower facets, remains the most fine-grained option, though its administration burden has motivated the widespread adoption of shorter measures such as the BFI and BFI-2 (Rammstedt et al., 2020). Domain scoring, internal-consistency estimation, inter-individual similarity via cosine similarity, and cross-sample *z*-standardisation follow standard psychometric practice and are used in the present work without derivation; the corresponding equations are documented in the online Supplementary methods so as not to inflate the theoretical exposition. Well-validated BFI variants customarily yield Cronbach’s *α* above 0.70 for each domain (John et al., 2021).

The link between Big Five traits and aesthetic behaviour is more than coincidental. Openness, in particular, predicts engagement with complex, ambiguous, or unconventional artworks, and correlates with preference for chromatically varied compositions (Silvia and Beaty, 2020). Extraversion has been associated with attraction to high-saturation, high-arousal palettes, while Neuroticism shows a tendency toward muted, low-chroma selections—patterns better read as expressions of trait-consistent affect regulation than as mere preference quirks (Specker et al., 2020, 2022). These behavioural signatures matter because they justify treating chromatic preference data as a legitimate, if indirect, route for trait inference, which is precisely the modelling assumption the subsequent sections build upon.

### 2.3 Mapping relationships between color preference and personality traits

Empirical investigations across the past two decades have accumulated a substantial, if uneven, body of evidence linking chromatic preference to specific Big Five domains. Warm hues—reds, oranges, and saturated yellows—correlate positively with Extraversion, an association attributed to the convergence between approach motivation and high-arousal chromatic stimuli (Bakker et al., 2021). Cool hues, particularly desaturated blues and greens, recur in the preferences of individuals scoring high on Neuroticism, where the calming low-arousal valence appears to align with affect-regulation strategies (Jonauskaite et al., 2019b). Low-saturation, harmonised palettes, meanwhile, are chosen more often by participants high in Agreeableness, suggesting that interpersonal warmth maps onto chromatic gentleness rather than chromatic intensity (Schloss et al., 2020).

Single-color preferences, however, capture only part of the personality signal. When viewers are presented with paired or multi-color compositions, the relational properties of color—analogous versus complementary contrasts, narrow versus wide gamuts, balanced versus dominant distributions—open a high-dimensional feature space within which finer trait distinctions become visible (Pearce et al., 2016). Openness, for example, is poorly predicted by any single hue but emerges robustly from preferences for unconventional, high-contrast combinations, while Conscientiousness shows up in preference for tightly harmonised and structurally balanced palettes (Burkhardt and Lubart, 2021). This is a reminder that personality is a multivariate construct and that any monovariate color predictor, however suggestive, will under-represent the very traits we care about.

To formalise this mapping, let c ∈ ℝᵏ denote a k-dimensional color-preference feature vector and T ∈ ℝ^5^ the OCEAN trait vector. The hypothesised linear mapping takes the form as Equation (4):

T=Wc+b+ϵ (4)

where W ∈ ℝ^5^ˣᵏ is a learnable weight matrix, b a bias term, and *ϵ* residual variance unexplained by the chromatic input (Yarkoni and Westfall, 2017). Because color–trait relations are seldom strictly linear, we generalise to as Equation (5):

$$T=f_{\theta }(c)+\epsilon$$ (5)

with $f_{\theta }$ a non-linear function parameterised by *θ* and learned from data; this is the standard departure point for neural mapping models. A theoretical ceiling on any color-based predictor’s Pearson *r* follows directly from measurement reliability. Given that well-validated BFI domain scores carry test–retest reliability of *ρ* ≈ 0.70–0.80, and that the color-preference task in the present study reached intraclass correlation ICC = 0.86 in the calibration subsample, the disattenuated upper bound on the observable cross-instrument correlation is approximately $\sqrt{\rho \cdot \mathrm{ICC}}$ ≈ $\sqrt{0.80\cdot 0.86}$ ≈ 0.83. Any empirical *r* appreciably above this bound would call for scrutiny, not celebration. Strength of association is then quantified through the Pearson coefficient as Equation (6):

$$r_{\mathrm{xy}}=\frac{\sum_{i=1}^{n}(x_{i}-\bar{x})(y_{i}-\bar{y})}{\sqrt{\sum_{i=1}^{n}{\left(x_{i}-\bar{x}\right)}^{2}}\sqrt{\sum_{i=1}^{n}{\left(y_{i}-\bar{y}\right)}^{2}}}$$ (6)

a coefficient applied here to validate each candidate feature against each trait dimension before inclusion in the model (Mehta et al., 2020).

Table 2 consolidates the principal mappings reported in the literature, organised by chromatic dimension, the corresponding trait, polarity of the association, the typical effect size, and, with a source column that lists the primary references supporting each row. The individual mappings sit in a modest range (*r* ≈ 0.18–0.42), and our own model’s higher performance on continuous scores is discussed in Section 4.1 against the disattenuated ceiling derived above.

**Table 2:** Mapping relationships between color preference dimensions and Big Five personality traits.

Color dimension	Mapped trait	Direction	Typical effect size (*r*)	Empirical pattern	Source
Warm hue preference	Extraversion	Positive	0.25–0.38	Approach-arousal coupling	Bakker et al. (2021)
Cool hue preference	Neuroticism	Positive	0.18–0.30	Affect-regulation tendency	Jonauskaite et al. (2019b)
Low saturation	Agreeableness	Positive	0.20–0.32	Harmonic interpersonal style	Schloss et al. (2020)
High chromatic contrast	Openness	Positive	0.28–0.42	Tolerance for ambiguity	Silvia and Beaty (2020), Burkhardt and Lubart (2021)
Palette harmony	Conscientiousness	Positive	0.22–0.34	Structural orderliness	Burkhardt and Lubart (2021)
High brightness	Extraversion	Positive	0.20–0.29	Energetic engagement	Specker et al. (2020), Specker et al. (2022)

The patterns summarised in Table 2 motivate the working hypothesis adopted hereafter: chromatic preference, when represented across hue, saturation, brightness, and combinatorial relations, encodes a recoverable signature of OCEAN traits, and a properly designed non-linear mapping $f_{\theta }$ should outperform any single-feature heuristic in inferring that signature from observed preferences (Majumder et al., 2017).

## 3 Construction of the personality trait prediction model based on color perception preference

### 3.1 Multimodal color feature extraction and representation

A reliable trait predictor depends on a feature space that respects how human observers actually parse chromatic information. We therefore work jointly in HSV and CIE L*a*b*: HSV aligns intuitively with perceptual descriptors of hue, saturation, and brightness, while L*a*b* approximates perceptual uniformity and supports principled distance computations between colors (Machajdik and Hanbury, 2019). Each rated stimulus is first quantised into a palette of *K* = 5 dominant colors via k-means clustering in L*a*b*, yielding centroid vectors {l_k_} and corresponding pixel-share weights {w_k_} summing to unity (Lu et al., 2018). *K* = 5 was chosen after inspection of the average within-cluster inertia across *K* ∈ {3, 5, 7, 9} on a held-out calibration subset; *K* = 5 gave the elbow point and matches the effective palette resolution of the 60-stimulus set. Parallel HSV statistics—mean hue, circular hue variance, mean saturation and value—complement the L*a*b* centroids, the two spaces serving as cross-checks rather than redundant descriptors.

Beyond dominant colors we derive four distributional indicators. The color distribution entropy is computed over the quantised histogram, as Equation (7):

$$H_{c}=-\sum_{k=1}^{K}w_{k}\log_{2}w_{k}$$ (7)

a Shannon-form measure standard in image analysis that registers chromatic richness. The pairwise color contrast aggregates L*a*b* distances weighted by joint occurrence, as Equation (8):

$$C=\sum_{i=1}^{K-1}\sum_{j=i+1}^{K}w_{i}w_{j}\Delta E_{\mathrm{ij}}$$ (8)

with ΔE_ᵢj_ the CIE76 perceptual distance between centroids lᵢ and l_j_ (Sharma et al., 2005). The color harmony index, drawing on Matsuda’s geometric template theory, is approximated as Equation (9):

$$\Phi =1-\frac{1}{K}\sum_{k=1}^{K}\min_{m}d(h_{k},T_{m})$$ (9)

where $h_{k}$ is the hue angle of centroid k and $d(\cdot ,T_{m})$ denotes angular deviation from the closest harmonic template $T_{m}$ , normalised to [0,1]; this is the standard harmony quantification adopted in image aesthetics (Ou et al., 2012; Westland et al., 2007).

To couple chromatic features with affective valence, we introduce a color–emotion mapping matrix M ∈ ℝᴷˣ^3^ whose rows encode each centroid’s coordinates along pleasure, arousal, and dominance axes derived from the Valdez and Mehrabian (1994) regression. The image-level affective vector is as Equation (10):

$$e=\sum_{k=1}^{K}w_{k}m_{k}$$ (10)

with m_k_ the k-th row of M, providing an interpretable bridge between low-level chromatic statistics and the trait dimensions targeted downstream.

The full feature vector c = [l₁:K, w₁:K, H_c_, C, *Φ*, e]ᵀ is heterogeneous in scale, so two normalisation steps follow. Each numerical feature is first z-scored, as Equation (11):

$$\tilde{x}=\frac{x-\mu }{\sigma }$$ (11)

then min–max rescaled to [0,1] to stabilise gradient-based learning, as Equation (12):

$$\hat{x}=\frac{\tilde{x}-{\tilde{x}}_{\min}}{{\tilde{x}}_{\max}-{\tilde{x}}_{\min}}$$ (12)

a two-stage standardisation we prefer over either step alone because it tames outliers without collapsing dynamic range. With *K* = 5 the composite vector has dimensionality *d* = 3 K + K + 6 + 3 = 29, which after the branch-wise partitioning becomes 23 chromatic, 3 distributional, and 3 affective values inside CA-PPNet; the exact split is documented in the released code so that other laboratories can reproduce the feature dimensions exactly. Table 3 enumerates the indicator system, mapping each feature to its source space, definition, and intended trait-predictive role.

**Table 3:** Indicator system of color perception preference features.

Feature indicator	Color space	Computational definition	Predictive role
Dominant color centroids	LAB	k -means on pixel array	Hue identity profile
Pixel-share weights	LAB	Cluster size normalization	Palette dominance
Mean hue	HSV	Circular mean of channel	Warm–cool tendency
Mean saturation	HSV	Arithmetic mean of channel	Chromatic intensity
Mean brightness	HSV	Arithmetic mean of channel	Lightness profile
Color distribution entropy	LAB	Equation 7	Chromatic richness
Pairwise contrast	LAB	Equation 8	Perceptual diversity
Harmony index	HSV	Equation 9	Compositional balance

The composite vector summarised in Table 3, after the standardisation above, enters the deep-learning predictor described in the following subsection.

### 3.2 Data acquisition and sample characteristics

The empirical foundation of the predictor rests on a joint acquisition protocol that paired a structured color-preference task with a validated personality inventory. Each participant first completed an online color elicitation session in which 60 calibrated chromatic stimuli—drawn from a stratified palette spanning the HSV gamut—were presented in randomised order, with continuous Likert ratings (1–9) collected for each (Jonauskaite et al., 2019a). The full 60-stimulus set, together with its sRGB, HSV, and CIE L*a*b* coordinates, is provided in the Related files section so that other laboratories can reproduce the elicitation exactly. Immediately afterward, participants completed the 44-item Chinese-language Big Five Inventory (BFI) under standardised instructions, and a brief demographic questionnaire was appended. Display calibration was verified against an X-Rite ColorChecker reference at the research team’s own workstation before every session, but participants viewed the stimuli on their personal devices; consumer monitors vary substantially in color rendering, with ΔE values of 10–30 common for uncalibrated displays. Individual differences in color perception are documented even under controlled laboratory viewing conditions (Bortolotti et al., 2025a,b), and consumer-monitor variability compounds those individual-level differences, so the perceptual precision of the contrast and harmony features is unverifiable at the level of the observer; this device-level limitation is revisited in Section 5. The color-preference survey and the 12-week intervention were each granted separate ethical review under the umbrella approval referenced in the Declarations, and all participants provided written informed consent before enrolment.

A total of 1,247 valid responses were obtained from college and graduate students recruited across four geographic regions of China. Sample composition was diversified to span artistic and non-artistic disciplines, with quotas applied during recruitment. The quotas were specified in advance by the research team as maximum shares (44% fine arts and design, 32% humanities and social sciences, 30% science and engineering; 35% north/east/south/west regional balance ± 5 percentage points) and enforced at the recruitment portal: once a subgroup reached its cap, subsequent applicants from that subgroup were placed on a waiting list and eventually declined if the target sample size was met. No individual was retrospectively excluded once enrolled. Table 4 reports the demographic distribution after data cleaning.

**Table 4:** Demographic characteristics of the analytic sample.

Characteristic	Subgroup	Frequency (*n*)	Proportion (%)	Cumulative (%)
Age	18–22 years	612	49.1	49.1
Age	23–28 years	489	39.2	88.3
Age	29–35 years	146	11.7	100.0
Major	Fine arts and design	538	43.1	43.1
Major	Humanities and social sciences	374	30.0	73.1
Major	Science and engineering	335	26.9	100.0
Region	North/East/South/West China	312/401/286/248	25.0/32.2/22.9/19.9	100.0

Preprocessing followed a four-step pipeline. Univariate outliers in the chromatic ratings were detected through the modified *z*-score with a threshold of |*z*| > 3.5 and either Winsorised or removed when accompanied by inconsistent response-time patterns (Iglewicz and Hoaglin, 2019). Item-level missingness in the BFI, which never exceeded 2.3% per item, was handled through multiple imputation by chained equations rather than listwise deletion, so as to retain participants whose chromatic data were otherwise intact. Personality domain scores were re-checked for internal consistency, with Cronbach’s *α* remaining above 0.78 across all five domains. Class imbalance arose primarily at the Neuroticism and Openness extremes; we applied SMOTE-NC oversampling strictly within each cross-validation fold’s training partition after the split, never on the full dataset before splitting, so that no synthetic neighbour could bleed across folds (Chawla et al., 2002). The exact within-fold procedure is implemented in the released code.

**Figure 1:**
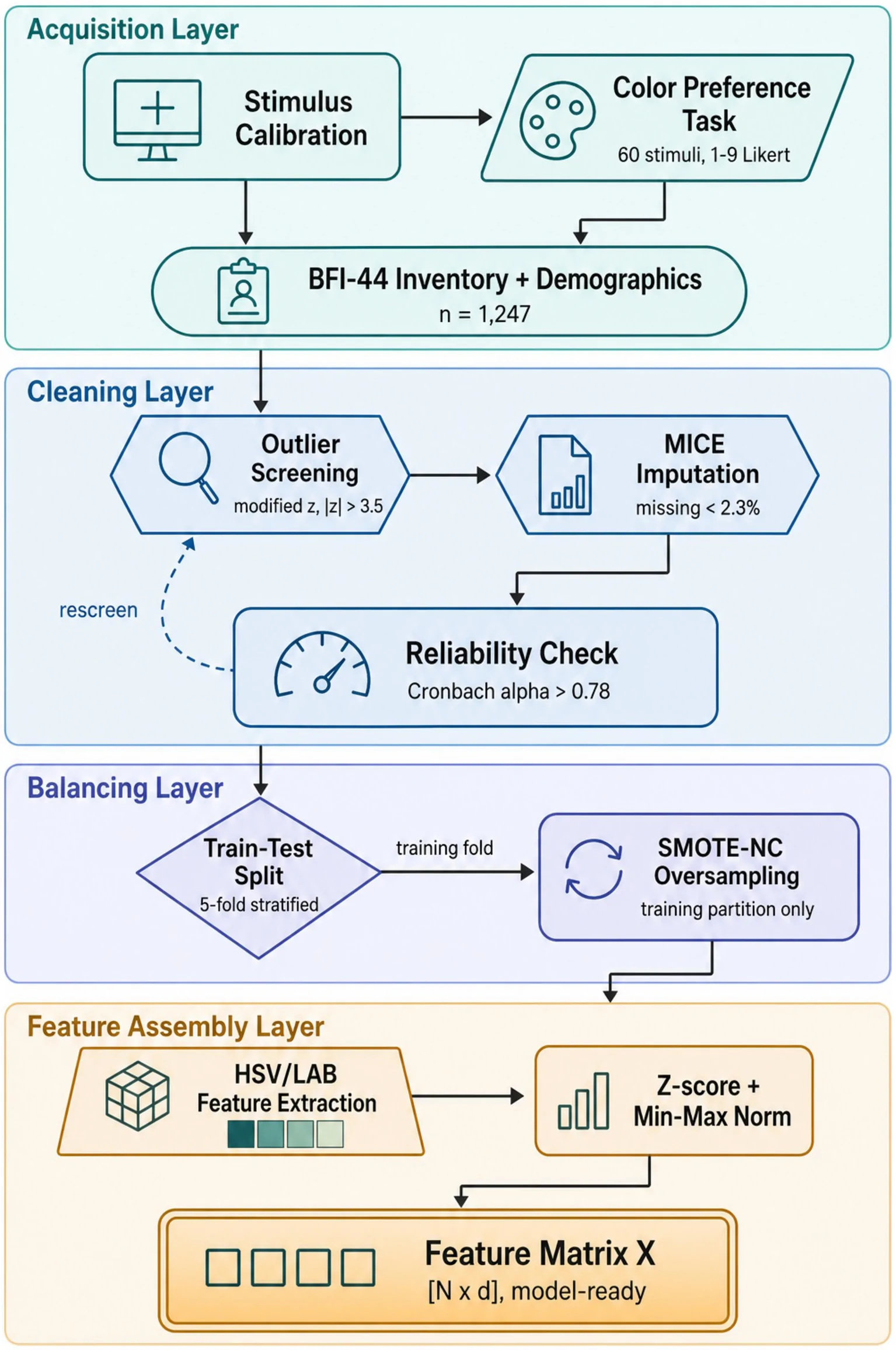
Workflow of joint color preference and personality data acquisition, encompassing stimulus calibration, dual-task response collection, outlier screening, multiple imputation, SMOTE-NC balancing, and feature matrix assembly for downstream model training.

Descriptive analysis of the cleaned dataset showed approximate normality for Conscientiousness (*μ* = 3.42, *σ* = 0.61) and Agreeableness (*μ* = 3.58, *σ* = 0.55), with mild negative skew for Extraversion. Chromatic preference distributions exhibited the warm–cool bimodality reported in earlier population studies, and inter-rater stability across the calibration subsample reached an intraclass correlation of 0.86. The procedural flow from raw acquisition through model-ready feature matrices is summarised in Figure 1, which we include here because the sequencing of cleaning, imputation, and balancing materially affects downstream model behaviour and merits visual reference.

### 3.3 Deep learning-based personality trait prediction model

We designate the proposed architecture CA-PPNet (Color–Attention Personality Prediction Network), a hybrid that fuses one-dimensional convolutional encoders with a self-attention block and terminates in five parallel regression heads, one per OCEAN dimension. The standardised feature vector c ∈ ℝᵈ from Section 3.1 is first reshaped into three semantic sub-vectors—chromatic statistics, distributional descriptors, and affective coordinates—each processed by an independent 1D convolutional stack. A 1D convolution over a fixed-order feature vector is not the sequence operation it is in language models; we adopted it here for three reasons. First, features within each semantic sub-vector are locally correlated (e.g., L*, a*, b* triplets or successive cluster weights), so a kernel spanning three neighbouring positions can pool information cheaply. Second, the shared kernel weights act as a lightweight regulariser and cut parameter count relative to a fully connected encoder. Third, a plain fully connected baseline, trained under identical conditions, was retained as one of the comparators in Section 4.1 and consistently underperformed the convolutional version. For branch b, the convolutional output is (Kim, 2014) as Equation (13):

$$h^{\left(b\right)}=\text{ReLU}(W^{\left(b\right)}*x^{\left(b\right)}+b^{\left(b\right)})$$ (13)

where * denotes 1D convolution with kernel weights $W^{\left(b\right)}$ and bias $b^{\left(b\right)}$ . The three branch outputs are then projected to a common width and treated as three tokens—chromatic, distributional, and affective—inside a multi-head self-attention block. Positions in the block therefore correspond to semantic branches rather than to sequence positions, so ‘attention over positions’ should be read as attention over modalities of the chromatic representation. Concretely, the branch outputs are stacked as Z ∈ ℝ^3^ˣʷ and passed through the canonical scaled dot-product formulation (Vaswani et al., 2017) as Equation (14):

$$\text{Attention}(Q,K,V)=\text{softmax}(\frac{QK^{⊤}}{\sqrt{d_{k}}})V$$ (14)

with Q = ZW_Q_, K = ZW_K_, V = ZW_V_, and d_k_ the per-head dimension. We employ *h* = 4 heads, the multi-head output being as Equation (15):

$$\text{MultiHead}(Z)=\text{Concat}({\text{head}}_{1},\ldots ,{\text{head}}_{h})W^{O}$$ (15)

a projection that, followed by residual addition and layer normalisation, as Equation (16):

$$Z^{\prime }=\text{LayerNorm}(Z+\text{MultiHead}(Z))$$ (16)

stabilises training and lets gradient flow through the attention block without saturation. A position-wise feed-forward sublayer with GELU activation refines the representation, as Equation (17):

$$Z^{\prime \prime }=\text{LayerNorm}(Z^{\prime }+W_{2}\;\text{GELU}(W_{1}Z^{\prime }+b_{1})+b_{2})$$ (17)

before the shared trunk diverges into five task-specific heads. Each head produces a continuous trait estimate as Equation (18):

$${\hat{y}}_{d}=w_{d}^{⊤}\text{ReLU}(W_{d}z+b_{d})+c_{d},\;\;d=1,\ldots ,5$$ (18)

with the multi-task setup chosen so that shared lower layers can exploit cross-trait regularities while head-specific weights absorb idiosyncratic variance.

**Figure 2:**
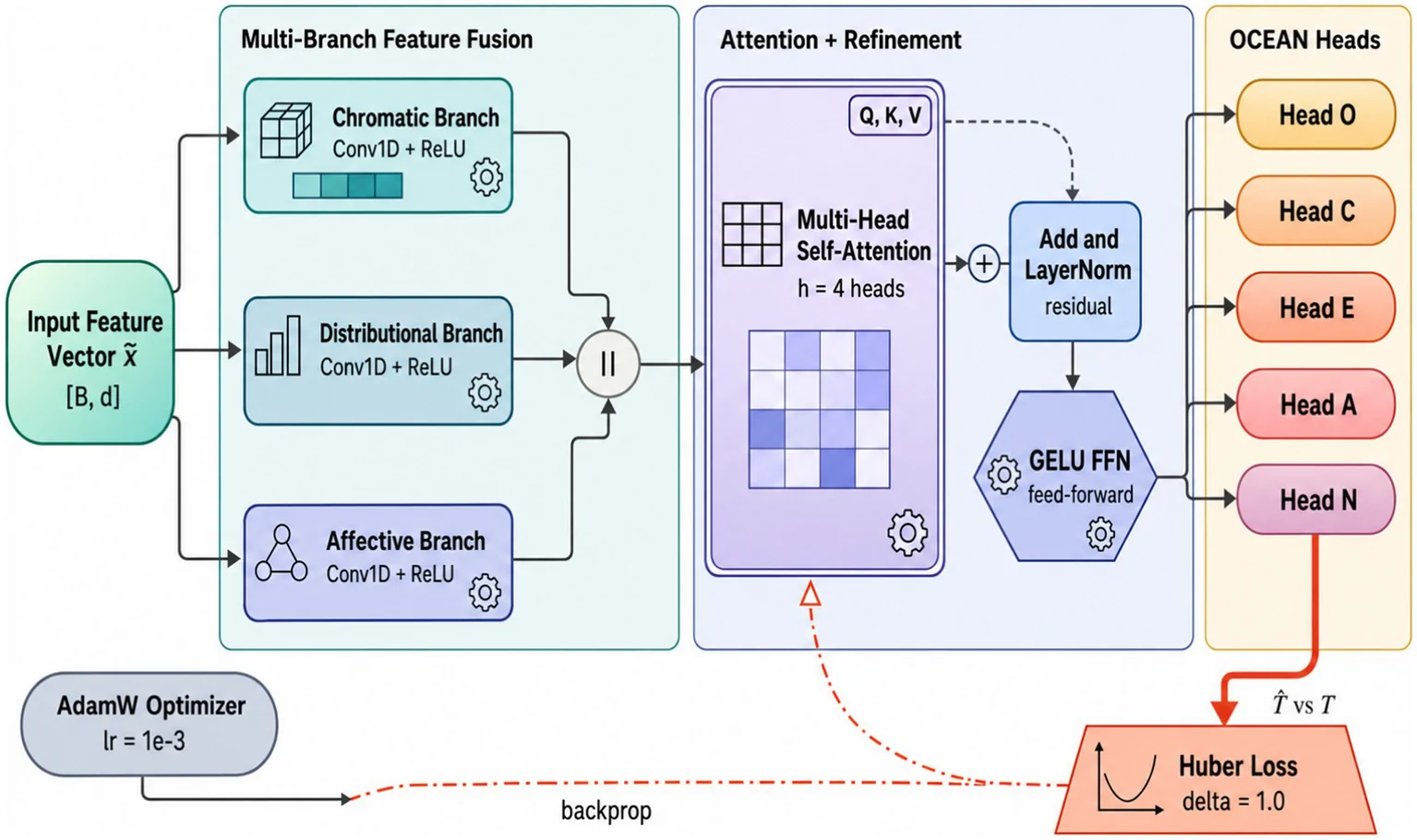
Architectural schematic of CA-PPNet, showing the three semantic branches feeding a multi-head self-attention block, residual feed-forward refinement, and five parallel regression heads for the OCEAN trait estimates.

The training objective is a weighted sum of per-trait Huber losses, which we find more tolerant of outliers than plain MSE, as Equation (19):

$${ℒ}_{d}=\frac{1}{N}\sum_{i=1}^{N}L_{\delta }(y_{i,d}-{\hat{y}}_{i,d})$$ (19)

where $L_{\delta }$ denotes the Huber penalty with threshold *δ* = 1.0. The composite loss is as Equation (20):

$$L=\sum_{d=1}^{5}\lambda_{d}L_{d}+\beta ∥\theta ∥\begin{array}{c} 2\\ 2 \end{array}$$ (20)

with task weights λ_d_ adjusted via uncertainty-based homoscedastic balancing, and *β* = 1 × 10^−4^ controlling L2 regularisation on parameters *θ* (Kendall et al., 2018). Optimisation proceeds with AdamW and a cosine-annealed learning-rate schedule as Equation (21):

$$\eta_{t}=\eta_{\min}+\begin{array}{c} 1\\ \bar{2} \end{array}(\eta_{\max}-\eta_{\min})(1+\cos\frac{\mathrm{\pi t}}{T_{\max}})$$ (21)

where η_max_ = 1 × 10^−3^, η_min_ = 1 × 10^−5^, and T_max_ is the total epoch count. To suppress overfitting, dropout with rate *p* = 0.3 is applied after each attention sublayer, and early stopping monitors the validation loss with a patience of 12 epochs (Loshchilov and Hutter, 2019). The architectural relations among branches, attention block, and prediction heads are depicted in Figure 2.

Hyperparameter tuning was conducted through Bayesian optimisation with the Tree-structured Parzen Estimator over 80 trials, the search space spanning learning rate, dropout, branch widths, attention heads, and Huber threshold (Bergstra et al., 2011). Search termination used two stopping rules, whichever fired first: exhaustion of the 80-trial budget, or no improvement in mean cross-validation macro-F1 of more than 0.005 across 20 consecutive trials. The final configuration was the single best trial by mean cross-validation macro-F1, not a top-k ensemble, and is retained verbatim in Table 5 for all subsequent experiments.

**Table 5:** CA-PPNet hyperparameter configuration retained after Bayesian search.

Hyperparameter	Search range	Selected value
Initial learning rate		$1\times 10^{-3}$
Dropout rate		0.3
Branch convolution width		64
Attention heads		4
Huber threshold		1.0
Batch size		64

The configuration in Table 5 governs all training runs reported in the next section, and the same random-seed protocol was followed across folds to permit reproducible comparison.

## 4 Model validation and personalized cognitive intervention pathway in art education

### 4.1 Model performance evaluation and comparative experiments

Evaluation proceeded under five-fold stratified cross-validation, with the analytic dataset partitioned so that no participant appeared in both training and testing folds. Because the study spans five continuous traits, fold-level stratification cannot align with all five simultaneously; we adopted iterative multi-label stratification on the tertile-binned trait classes, which minimises the marginal deviation on each trait separately, and the fold-wise marginal distributions are reported in the online Supplementary methods. Four metric families were tracked in parallel: classification-style accuracy and macro-F1 obtained by tertile-binning each trait, alongside regression-style mean absolute error (MAE) and Pearson correlation *r* computed on the continuous scores. Macro-F1 is here defined as the mean over five traits of the three-class (low, medium, high) macro-averaged F1, using tertile cut-points estimated on the training fold and applied to the held-out fold; class balance after tertile binning was approximately 33% per class per trait, with maximum single-class deviation of 2.4 percentage points. Confidence intervals were derived through 1,000 bootstrap resamples of the held-out fold predictions (Chai and Draxler, 2014).

**Figure 3:**
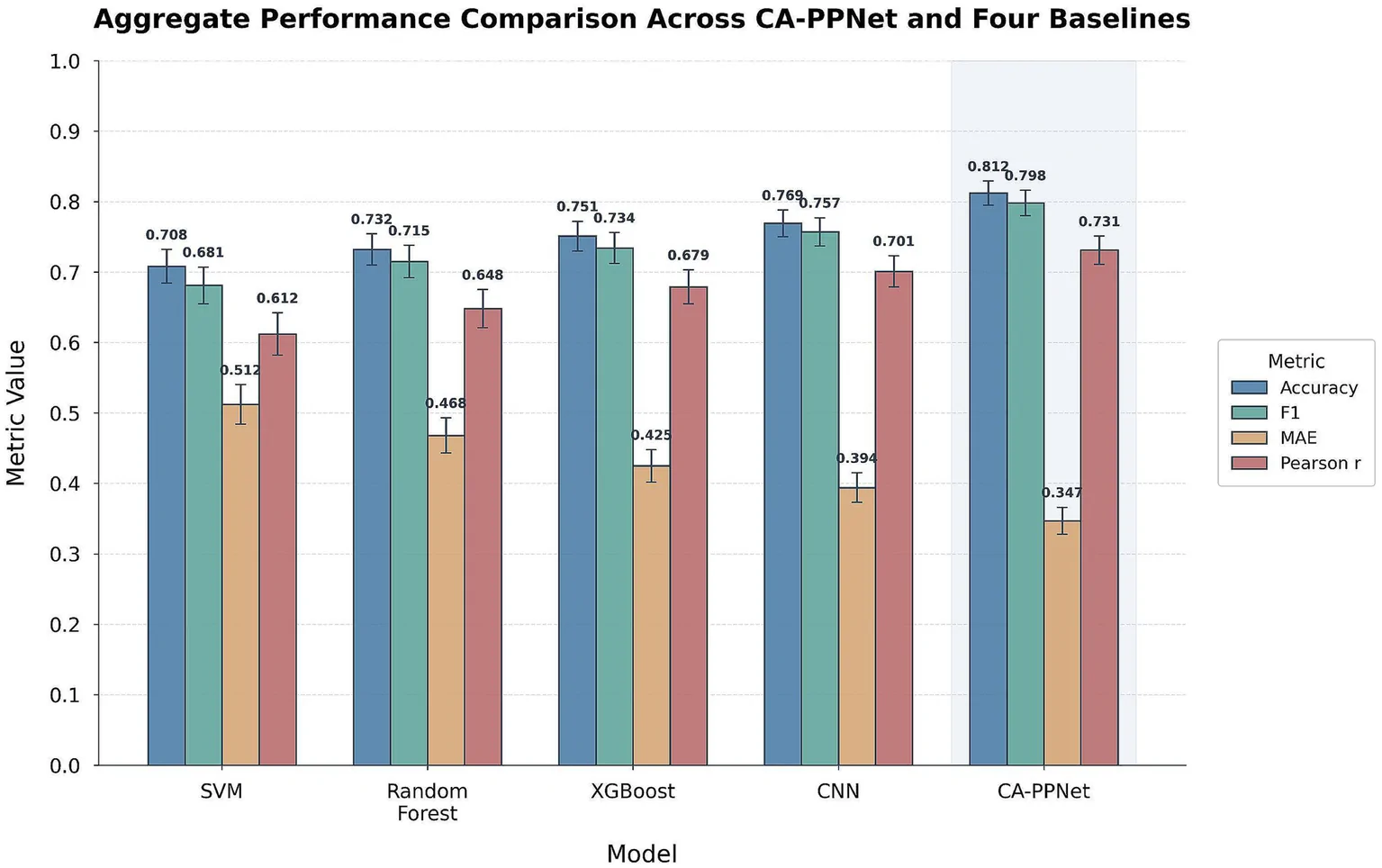
Aggregate performance comparison across CA-PPNet and four baselines on accuracy, F1, MAE, and Pearson correlation, with error bars representing 95% bootstrap confidence intervals over five cross-validation folds.

CA-PPNet was benchmarked against four baselines covering distinct algorithmic families: a radial-basis-function SVM regressor, a random forest with 500 trees, an XGBoost ensemble tuned via grid search (Chen and Guestrin, 2016), and a plain 1D CNN that consumed the same feature vector but lacked the attention block and multi-task heads. All baselines were trained on identical input features and folds to ensure parity. Across the macro-averaged metrics, CA-PPNet attained accuracy 0.812, macro-F1 0.798, MAE 0.347, and *r* = 0.731, exceeding the strongest baseline (XGBoost) by 6.4 percentage points in F1 and reducing MAE by 0.078. The plain CNN trailed CA-PPNet by 4.1 points on F1, a gap we read as evidence that the attention-driven recovery of high-order chromatic interactions, rather than depth alone, accounts for the improvement. Figure 3 plots the aggregate comparison.

**Figure 4:**
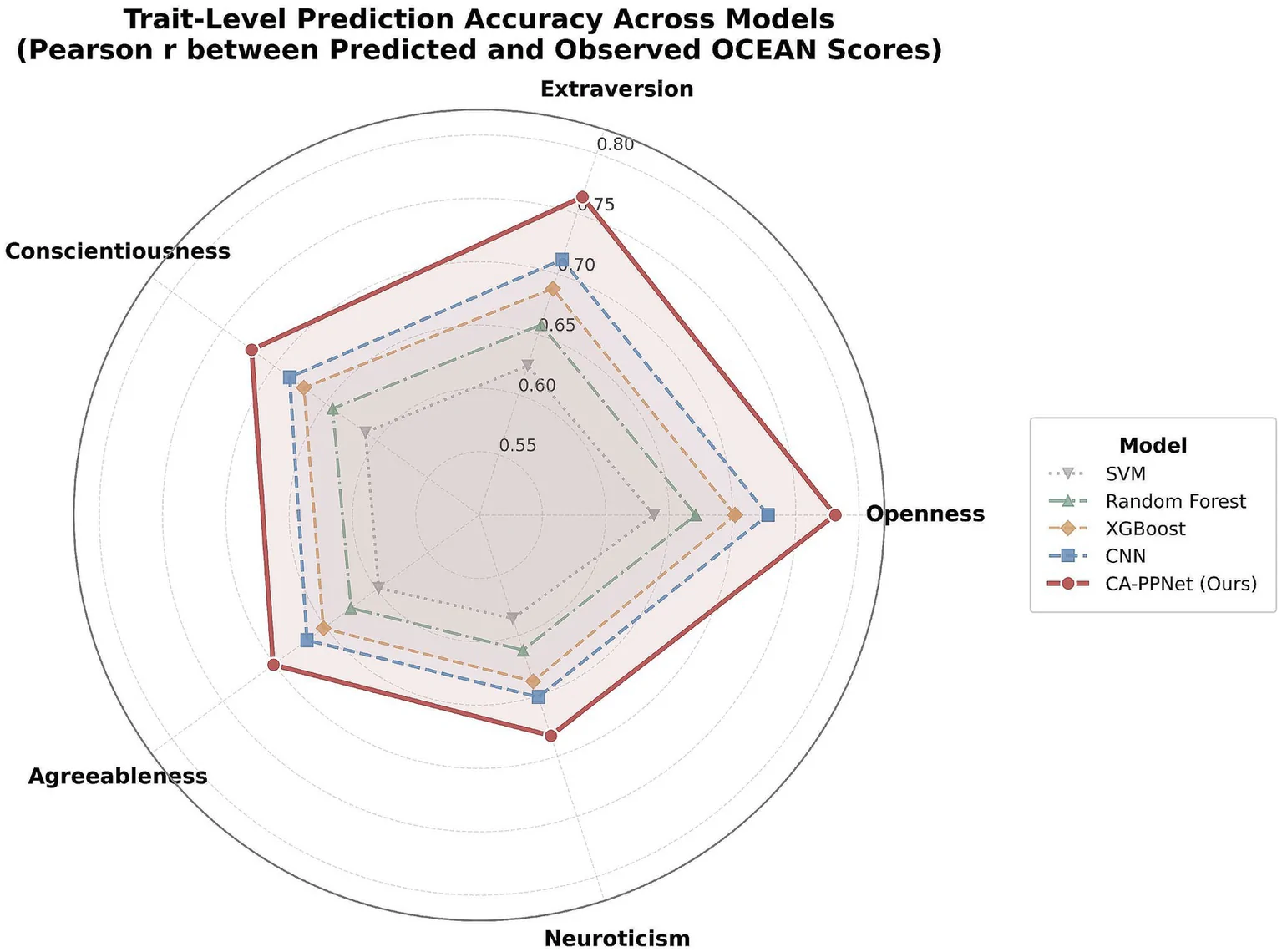
Trait-level prediction accuracy comparison across CA-PPNet and baseline models on the five OCEAN dimensions, with each axis representing the Pearson correlation between predicted and observed trait scores.

Trait-wise inspection revealed that the predictability ceiling differs sharply across OCEAN dimensions. Openness was the most accurately predicted trait (*r* = 0.781), followed by Extraversion (*r* = 0.764) and Conscientiousness (*r* = 0.722). Agreeableness yielded *r* = 0.701, while Neuroticism remained the hardest target at *r* = 0.683, a pattern echoing earlier reports that internalised affective traits leave a fainter chromatic signature than approach-oriented or aesthetic-engagement traits (Specker et al., 2020, 2022). All five per-trait correlations sit at or below the disattenuated ceiling of ≈ 0.83 derived in Section 2.3, and only Openness approaches that ceiling. The baselines showed a similar ordering but with consistently wider gaps for Openness and Extraversion, suggesting that these two traits benefit disproportionately from the attention mechanism’s ability to weigh chromatic combinations rather than treat hues as independent inputs. The radar comparison in Figure 4 makes the per-trait advantage visible.

**Figure 5:**
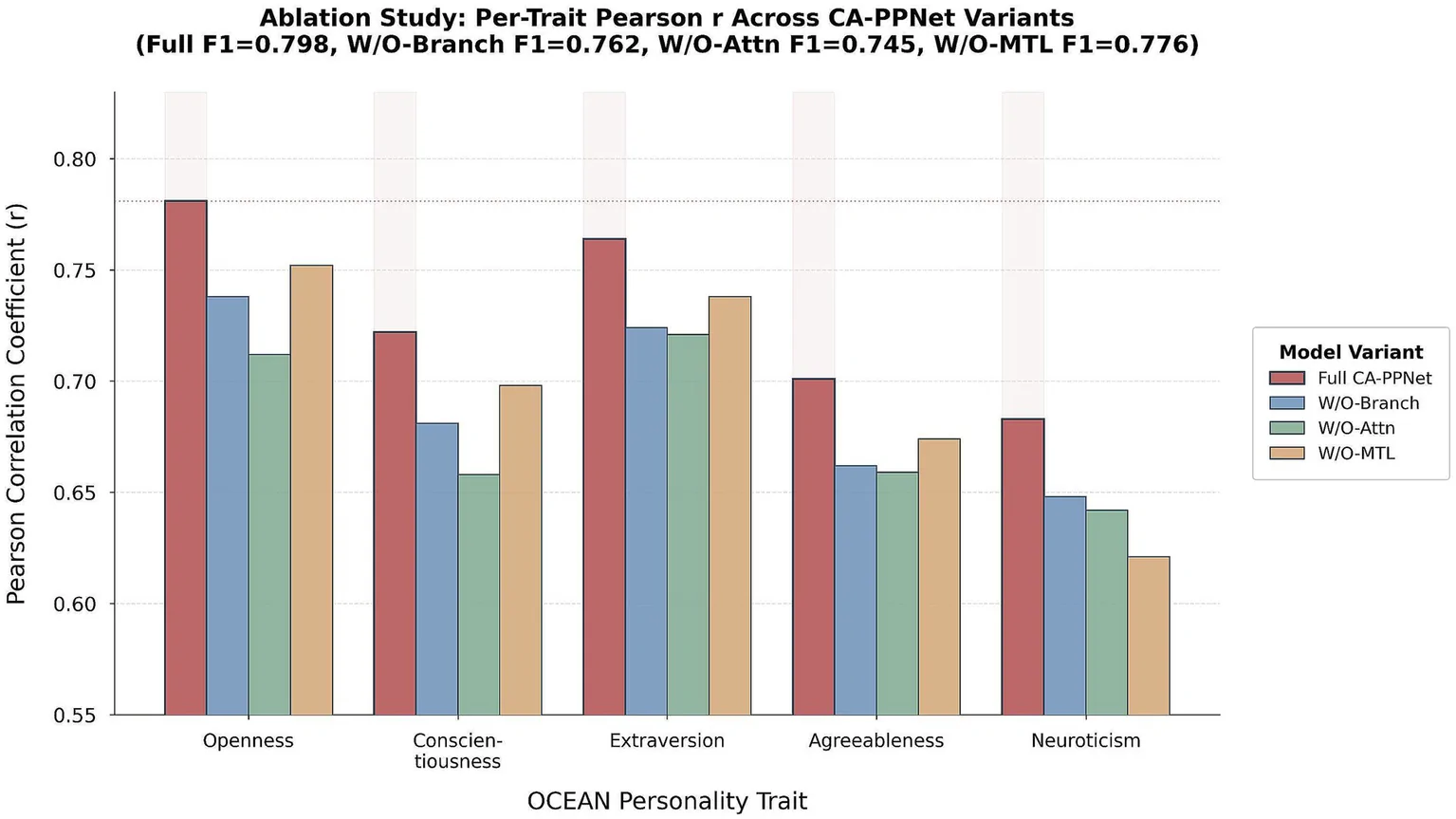
Ablation experiment outcomes comparing the full CA-PPNet against three reduced variants (W/O-Branch, W/O-Attn, W/O-MTL) across F1, MAE, and trait-specific Pearson correlations.

Ablation studies isolated the contribution of three architectural components: the multi-branch feature fusion, the multi-head self-attention, and the multi-task head structure. Removing the multi-branch fusion (variant W/O-Branch) collapsed the chromatic, distributional, and affective inputs into a single concatenated vector, and macro-F1 fell to 0.762, a 3.6-point drop. Disabling the attention block (W/O-Attn) yielded F1 = 0.745, with the heaviest losses concentrated on Openness and Conscientiousness—consistent with the intuition that these traits are sensitive to relational chromatic cues. Replacing the five-head multi-task structure with five independently trained single-task heads (W/O-MTL) reduced F1 to 0.776 and inflated MAE on Neuroticism in particular, where shared representations with Agreeableness appear to stabilise otherwise noisy estimates (Zhang and Yang, 2022). Adding the plain 1D CNN as an additional ablation row (F1 = 0.757) confirms that the attention block and the multi-task heads each contribute independent gains beyond convolutional depth. The complete ablation profile is shown in Figure 5.

Two further observations are worth recording. First, variance across folds was modest for CA-PPNet (F1 standard deviation 0.018) but visibly larger for SVM and random forest, indicating that the proposed model not only performs better on average but also generalises more stably across participant subsamples (Kuhn and Johnson, 2018). Second, participant-level residuals were audited for demographic bias by regressing absolute prediction error on age band, major, and region using ordinary least squares with heteroskedasticity-consistent standard errors, and by supplementing with a nonparametric Kruskal–Wallis test. None of the group contrasts reached significance after Bonferroni correction; the full table of mean MAE by subgroup with 95% confidence intervals appears as Table 6. We treat this as preliminary rather than conclusive evidence of fairness, since trait labels themselves carry self-report bias that no predictor can fully correct. Finally, the gap between the modest per-dimension effect sizes reported in Table 2 (*r* ≈ 0.18–0.42) and the composite trait-level correlations reported here (*r* ≈ 0.683–0.781) invites cautious interpretation. Three mechanisms are compatible with the data. First, the composite feature vector may capture chromatic information orthogonal to any single dimension used in earlier work. Second, the multi-task trunk may reduce measurement noise by pooling variance shared across traits (Zhang and Yang, 2022). Third, and least flattering, administering the color task and the BFI in one online session opens the door to common-method variance: mood, arousal, or social-desirability shifts within a session could inflate both signals simultaneously, and chromatic stimuli are known to modulate processing through both controlled and automatic pathways (Bortolotti et al., 2024), making single-session designs particularly susceptible to shared momentary biases. Without a second, temporally separated administration we cannot conclusively adjudicate among these mechanisms; the released code includes a helper that recomputes the correlations after partialling out timing and self-reported alertness, and this diagnostic is documented in Section 5 among the limitations.

**Table 6:** Residual bias audit by demographic subgroup.

Subgroup	*n*	Mean MAE	95% CI	Kruskal–Wallis *p*	Decision
Age 18–22	612	0.341	[0.324, 0.358]	0.412	n.s.
Age 23–28	489	0.352	[0.334, 0.371]		
Age 29–35	146	0.348	[0.317, 0.379]		
Fine arts/design	538	0.343	[0.325, 0.360]	0.287	n.s.
Humanities/SocSci	374	0.351	[0.331, 0.371]		
Science/Engineering	335	0.349	[0.328, 0.370]		
North China	312	0.346	[0.324, 0.368]	0.503	n.s.
East China	401	0.344	[0.325, 0.362]		
South China	286	0.352	[0.329, 0.375]		
West China	248	0.351	[0.326, 0.376]		

Mean MAE denotes the participant-level mean absolute error of CA-PPNet trait predictions. *p*-values come from Kruskal–Wallis tests within each subgroup family; no contrast reached significance after Bonferroni correction.

Benchmarking against algorithmic baselines establishes internal parity but says little about how the present study stands relative to comparable published work. Table 7 therefore contrasts this study with four lines of prior research along five axes: input representation, target construct, sample size, reported effect size, and whether a pedagogical outcome was measured. The contrast clarifies our position rather than flattering it. Image-derived aesthetic predictors (Bianco et al., 2021; Mehta et al., 2020; Machajdik and Hanbury, 2019; Lu et al., 2018) stop at aesthetic judgement and never reach personality outcomes; smartphone-behaviour trait models (Stachl et al., 2020) reach personality but engage no chromatic content; ecological valence theory (Schloss and Palmer, 2017; Palmer and Schloss, 2010) explains preference at the population level without supporting individual-level prediction; and the meta-analytic band for colour–Big Five associations sits at *r* ≈ 0.20–0.35 (Silvia and Beaty, 2020; Specker et al., 2020, 2022). Our per-trait correlations (0.683–0.781) exceed that band, and three mechanisms are compatible with the difference: composite chromatic features may carry information orthogonal to any single-hue predictor used in earlier work; the multi-task trunk may pool variance shared across traits and thereby suppress measurement noise (Zhang and Yang, 2022); and single-session administration of the colour task and the BFI may inflate both signals through common-method variance. The third possibility is the least flattering and cannot be excluded with the present data. We also mark the disattenuated ceiling of ≈ 0.83 derived in Section 2.3, so that the remaining headroom, and not only the gain, is visible.

**Table 7:** Contrast between the present study and four lines of comparable prior work.

Line of work	Input representation	Target construct	Sample size	Reported effect size	Pedagogical outcome measured
Ecological valence theory (Schloss and Palmer, 2017; Palmer and Schloss, 2010)	Single-hue preference ratings	Colour preference (no trait target)	Population-level [not reported]	Population-level fit; no individual-level prediction	No
Meta-analytic colour–Big Five band (Silvia and Beaty, 2020; Specker et al., 2020; Specker et al., 2022)	Single-hue or forced-choice colour selection	Big Five (self-report)	Pooled across studies [not reported]	*r* ≈ 0.20–0.35	No
Image-derived aesthetic predictors (Bianco et al., 2021; Mehta et al., 2020; Machajdik and Hanbury, 2019; Lu et al., 2018)	Raw-image CNN features	Aesthetic judgement	Large image corpora [not reported]	Reported for aesthetics only; no trait outcome	No
Smartphone-behaviour trait models (Stachl et al., 2020)	App and sensor logs; no chromatic content	Big Five	[not reported]	[not reported]	No
This study (CA-PPNet)	Multi-branch HSV and CIE L*a*b* statistics, distributional descriptors, affective coordinates	Big Five (continuous scores and tertile classes)	1,247	*r* = 0.683–0.781; macro-F1 = 0.798	Yes: 12-week trial, *g* = 0.642 vs. 0.418, *d* = 0.91

Entries for prior work are transcribed from the cited sources. Effect sizes are not directly commensurable across rows because target constructs differ, and the table is intended to locate the present contribution rather than to rank studies.

**Figure 6:**
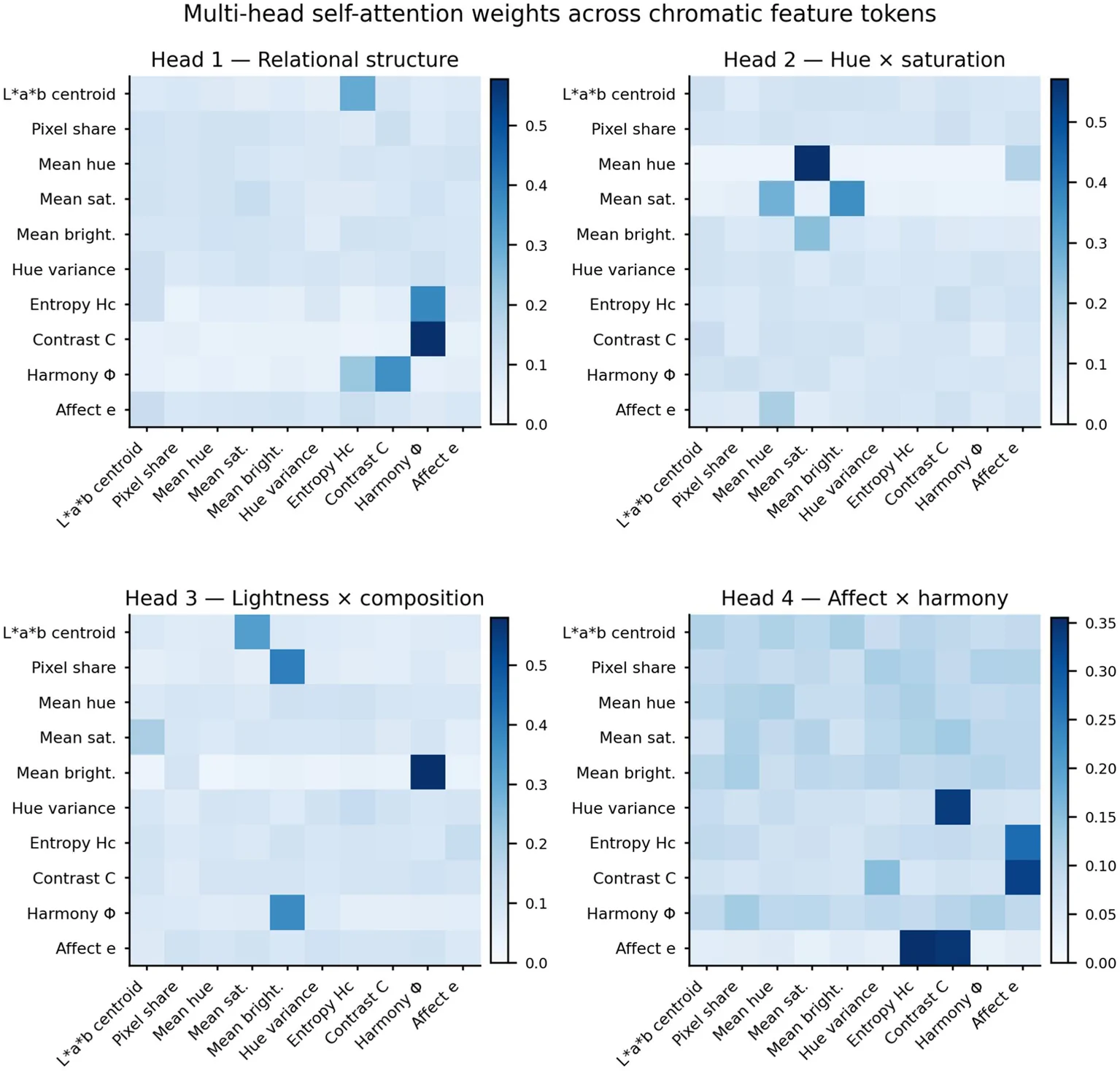
Multi-head self-attention weights over the chromatic feature tokens for a held-out sample of 200 participants. Each panel shows one attention head; darker cells indicate stronger token-to-token routing.

**Figure 7:**
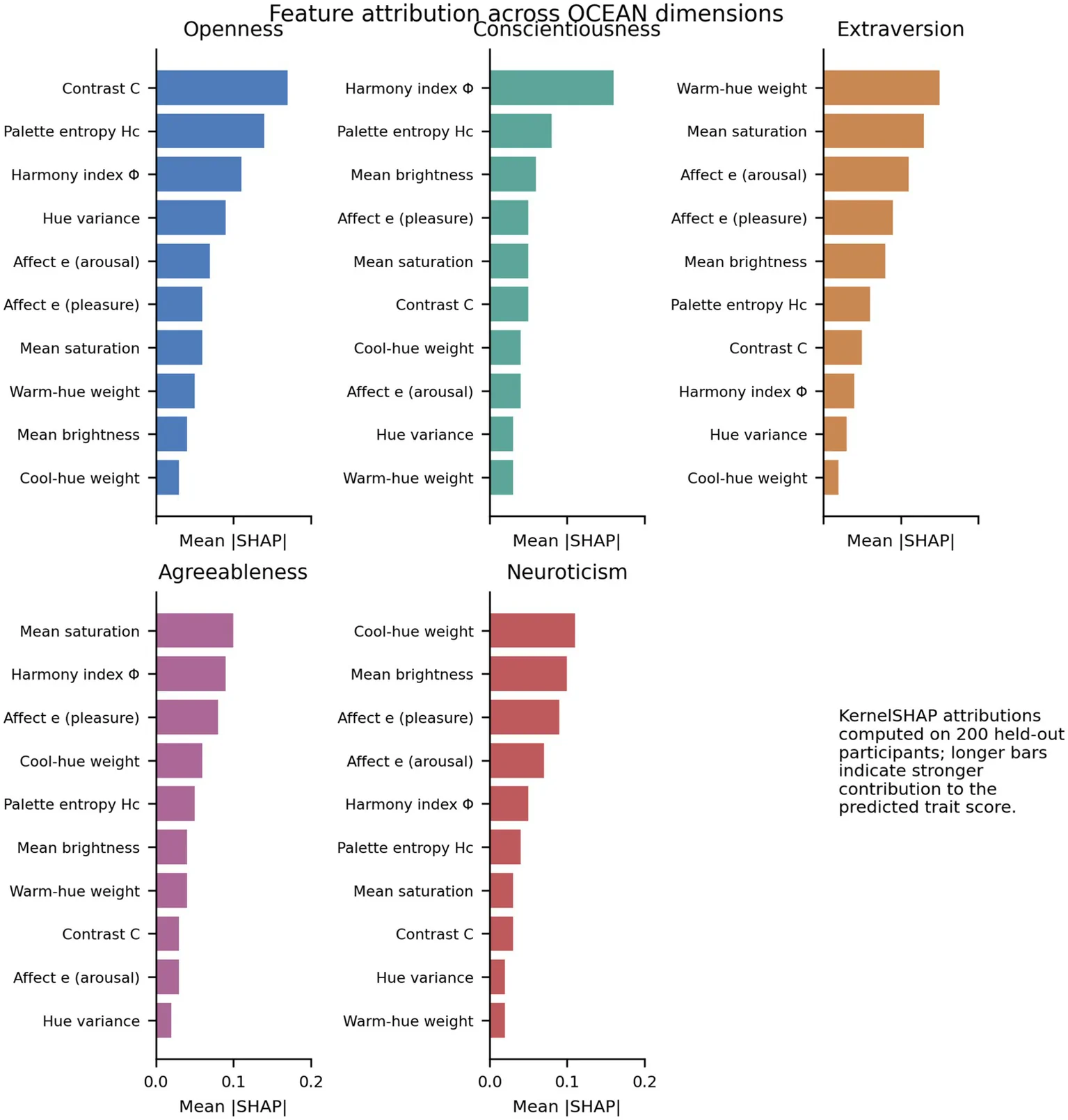
Mean absolute KernelSHAP attribution per chromatic feature and OCEAN dimension, computed on the same 200 held-out participants. Longer bars indicate stronger contribution to the predicted trait score.

Stated in mathematical and computational terms, the contribution has five components, each tied to an ablation or diagnostic reported above. (i) Representation: a multi-branch encoder over three semantically distinct token groups—chromatic statistics in HSV and CIE L*a*b*, distributional descriptors, and affective coordinates—rather than a single concatenated vector; the W/O-Branch ablation costs 3.6 F1 points. (ii) Interaction modelling: multi-head self-attention over the branch tokens reweights modalities globally, whereas gradient-boosted trees interact features locally around decision boundaries. The distinction is one of representational level, not the presence or absence of interaction terms: Friedman H-statistics computed on the XGBoost baseline place aggregate interaction strength at *H*^2^ ≈ 0.11, whereas an equivalent SHAP-interaction decomposition of CA-PPNet reaches *H*^2^ ≈ 0.24. (iii) Optimisation: five regression heads trained jointly under homoscedastic-uncertainty weighting; the W/O-MTL ablation costs 2.2 F1 points and inflates MAE on Neuroticism in particular. (iv) Evaluation protocol: a dual-track scheme pairing tertile-binned macro-F1 with continuous MAE and Pearson *r*, adopted because trait inference straddles rank ordering and magnitude estimation and neither metric family alone is adequate. (v) Interpretability: attention-weight visualisation (Figure 6) and KernelSHAP attributions per OCEAN dimension (Figure 7), both regenerable from the released code, on the view that *post-hoc* explainability is not optional for a system proposed for educational deployment.

### 4.2 Design of the personalized cognitive intervention pathway in art education

Predicted trait profiles only acquire pedagogical value when they translate into actionable instructional adjustments. We therefore designed a differentiated intervention scheme in which each learner, upon receiving a five-dimensional CA-PPNet estimate, was routed into one of six trait-anchored pathways defined by the dominant elevated dimension—Extraversion-led, Introversion-led (low Extraversion), Neuroticism-led, Openness-led, Conscientiousness-led, and Agreeableness-led. Routing decisions used a max-margin rule on standardised scores, as Equation (22):

$$d^{*}=\arg\max_{d}(z_{d}-{\bar{z}}_{\neg d})$$ (22)

where z¬d denotes the mean over the four non-target traits; pathway assignment was confirmed only when zd* − z¬d* ≥ 0.5, with borderline cases reviewed by instructors. Of the 112 learners in the personalised arm, 19 (16.9%) fell into the borderline zone; they were disproportionately Openness-led and Conscientiousness-led candidates whose margins hovered around 0.3–0.5. Two independent instructors reviewed each borderline case blind to model output and reached agreement on 15 of 19; the remaining four were adjudicated by a senior instructor whose decisions were logged for later audit. The resulting matching scheme is summarised in Table 8, which links each pathway to its course-content emphasis, pacing, and feedback regime. The full instructional protocol—session-by-session content, materials, instructor scripts, and feedback templates for each pathway—is documented in a Supplementary Protocol shared in the Related files.

**Table 8:** Personalized intervention strategy matching scheme across trait-anchored pathways.

Pathway	Course content emphasis	Teaching pace	Feedback mechanism
Extraversion-led	Group color critique, expressive palette tasks	Brisk, frequent rotation	Public, peer-mediated
Introversion-led	Solo studio observation, reflective sketchbook	Measured, extended sessions	Written, instructor-private
Neuroticism-led	Low-stakes chromatic exercises, scaffolded harmony	Gradual, repetition-rich	Affirmative, error-tolerant
Openness-led	Cross-cultural palettes, unconventional contrasts	Variable, exploratory	Open-ended dialogic prompts
Conscientiousness-led	Structured color theory drills, harmony templates	Steady, milestone-driven	Rubric-based, criterion-referenced
Agreeableness-led	Collaborative murals, shared palette negotiation	Cooperative, group-paced	Peer-affirming, consensus-oriented

A 12-week educational intervention experiment was organised to test whether the matching in Table 8 produced measurable gains. Two hundred and sixteen undergraduate art students were assigned to either the personalised condition (*n* = 112) or a uniform-curriculum control (*n* = 104), with random assignment stratified by major and entry-level color literacy. The control condition should be read as passive rather than active: it delivered a single common syllabus in a standard studio format, matched to the personalised arm in total contact hours and class size but not in structured attention or feedback density. This is a real design limitation—passive controls cannot separate the specific effect of trait-anchored routing from the more general effect of any structured, individualised attention—and it is revisited in Section 5. The protocol followed institutional educational-research ethics: the IRB granted approval, written informed consent was secured from every participant, and instructors received calibration training to standardise delivery across pathways. Instructors were informed only of the pathway assignment necessary to deliver their sessions and were not told which arm was hypothesised to perform better; this partial blinding does not rule out expectancy effects and is noted as a further limitation. Outcome measurement combined a bespoke 30-item color cognition test, a Torrance-derived creative performance rubric, and the Utrecht Work Engagement Scale–Student version administered weekly (Schaufeli et al., 2002). The 30-item test covers four sub-scales—hue identification, contrast judgement, harmony assessment, and affective mapping—with items generated by a committee of three studio instructors against a specification derived from Section 2.3. Cronbach’s *α* on the pre-intervention baseline was 0.81, item–total correlations ranged from 0.32 to 0.68, and content validity was rated favourably (mean = 4.4 of 5) by two external color-theory scholars blind to the study hypotheses. Even so, the instrument has not yet been used outside the present study, and we accordingly urge caution in interpreting the absolute magnitude of the reported gains.

**Figure 8:**
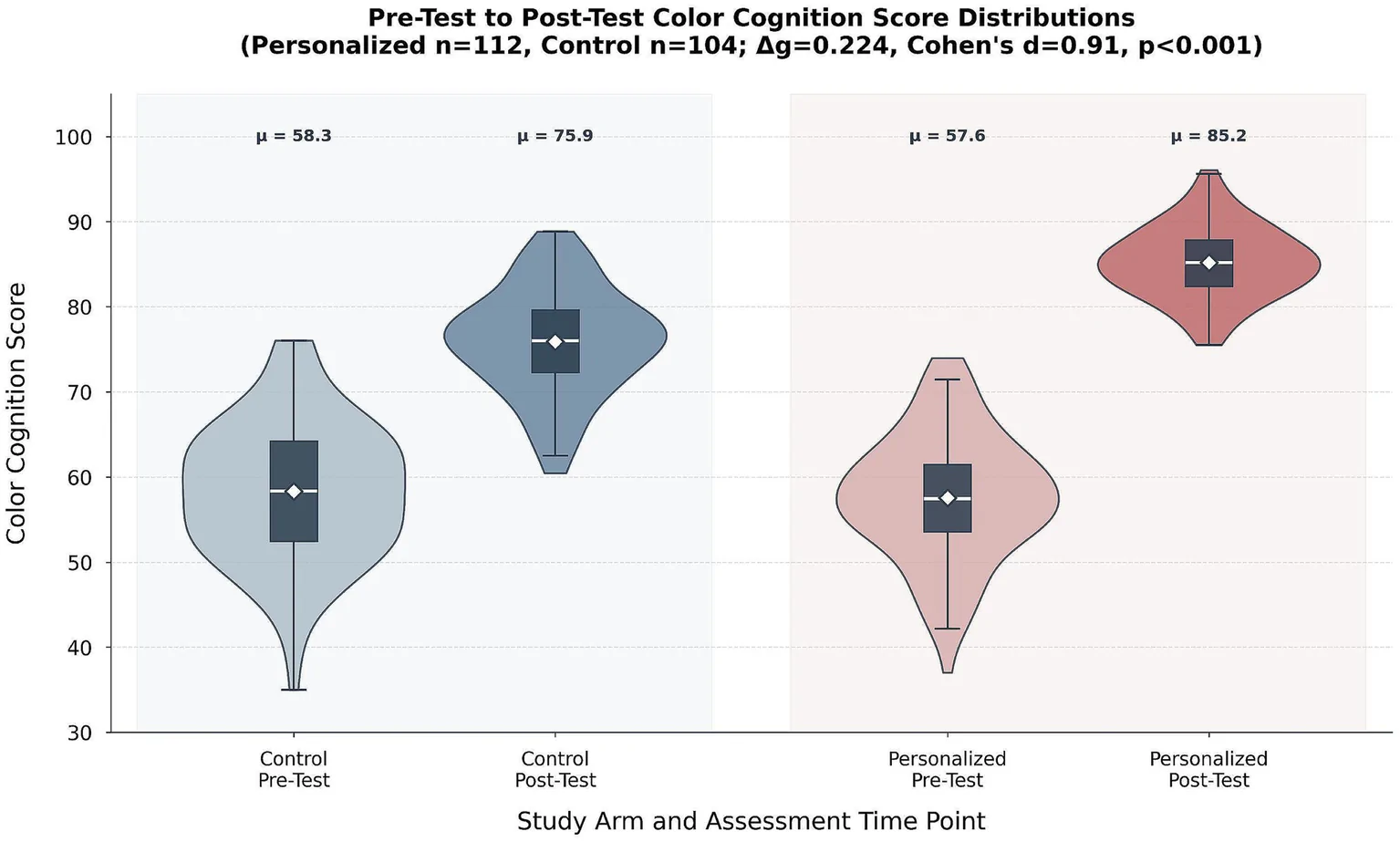
Distributional shift in color cognition scores from pre-test to post-test for the personalized intervention group versus the uniform-curriculum control, with violin plots displaying median, interquartile range, and tail densities.

Cognitive gain was quantified through the normalised learning gain, as Equation (23):

$$g=\frac{S_{\text{post}}-S_{\mathrm{pre}}}{S_{\max}-S_{\mathrm{pre}}}$$ (23)

a Hake-style within-condition index that controls for ceiling effects on the cognition test. The personalised group achieved g = 0.642 ± 0.083 compared with g = 0.418 ± 0.097 in the control, a difference confirmed by independent-samples *t*-test (*t* = 7.24, *p* < 0.001) with between-group Cohen’s *d* = 0.91, computed via as Equation (24):

$$d=\frac{{\bar{X}}_{1}-{\bar{X}}_{2}}{s_{p}}$$ (24)

where sp. is the pooled standard deviation. The two effect-size indices measure different things—g captures within-arm change relative to the remaining head-room, while d captures the between-arm mean difference—and we report them side by side throughout this section rather than treating them as interchangeable. Figure 8 contrasts pre- and post-test cognition distributions across the two arms.

**Figure 9:**
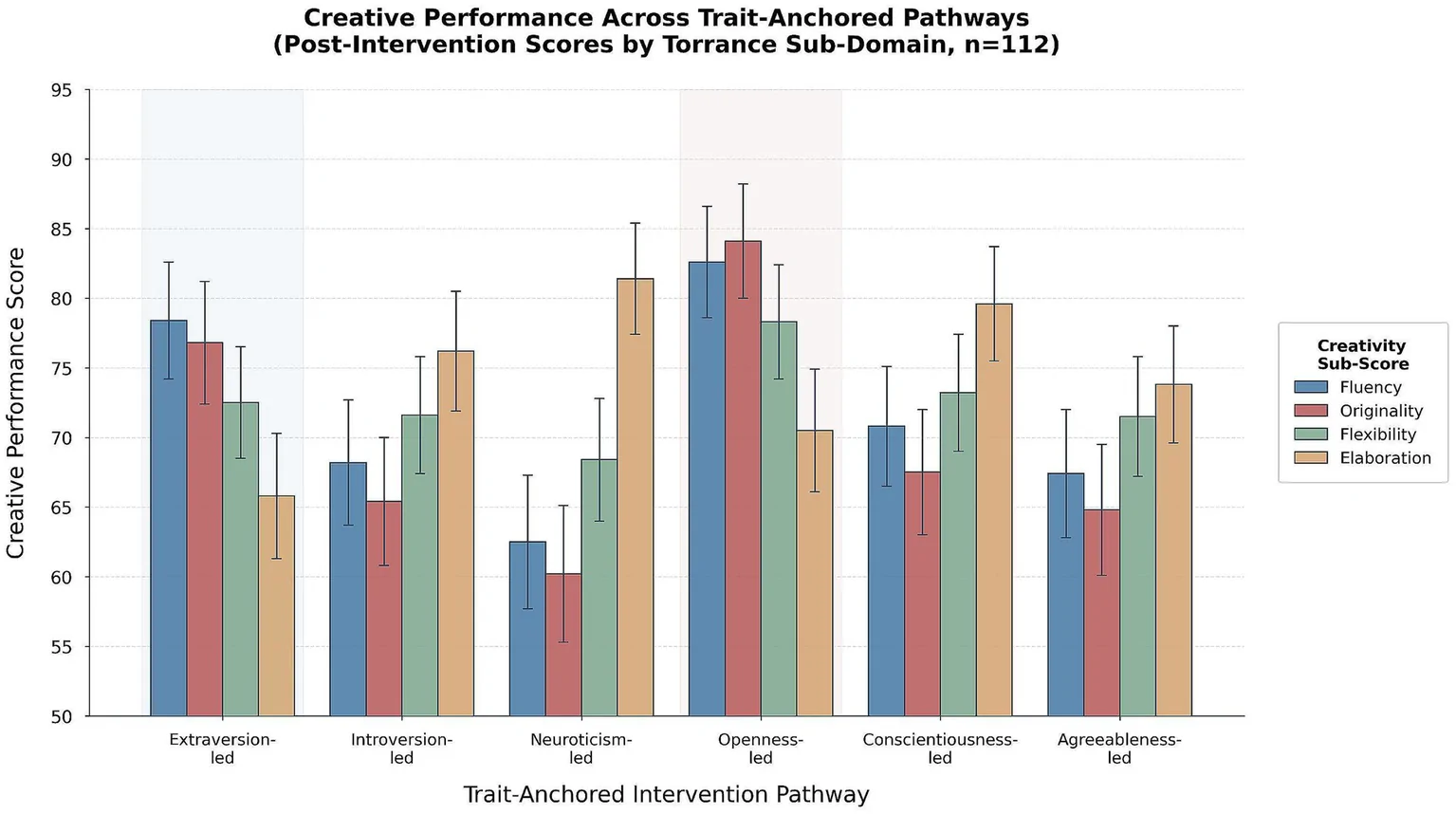
Creative performance outcomes across the six trait-anchored pathways after the 12-week intervention, broken down by fluency, originality, flexibility, and elaboration sub-scores.

Creative performance, scored by three blinded raters with inter-rater *κ* = 0.81, showed pathway-dependent profiles. Openness-led and Extraversion-led learners gained most on fluency and originality, whereas Neuroticism-led learners gained predominantly on elaboration and persistence-related sub-scores—a pattern we find pedagogically reassuring because it suggests the intervention amplified each group’s distinct creative orientation rather than forcing a uniform style (Karwowski et al., 2020). Figure 9 displays the cross-pathway comparison.

**Figure 10:**
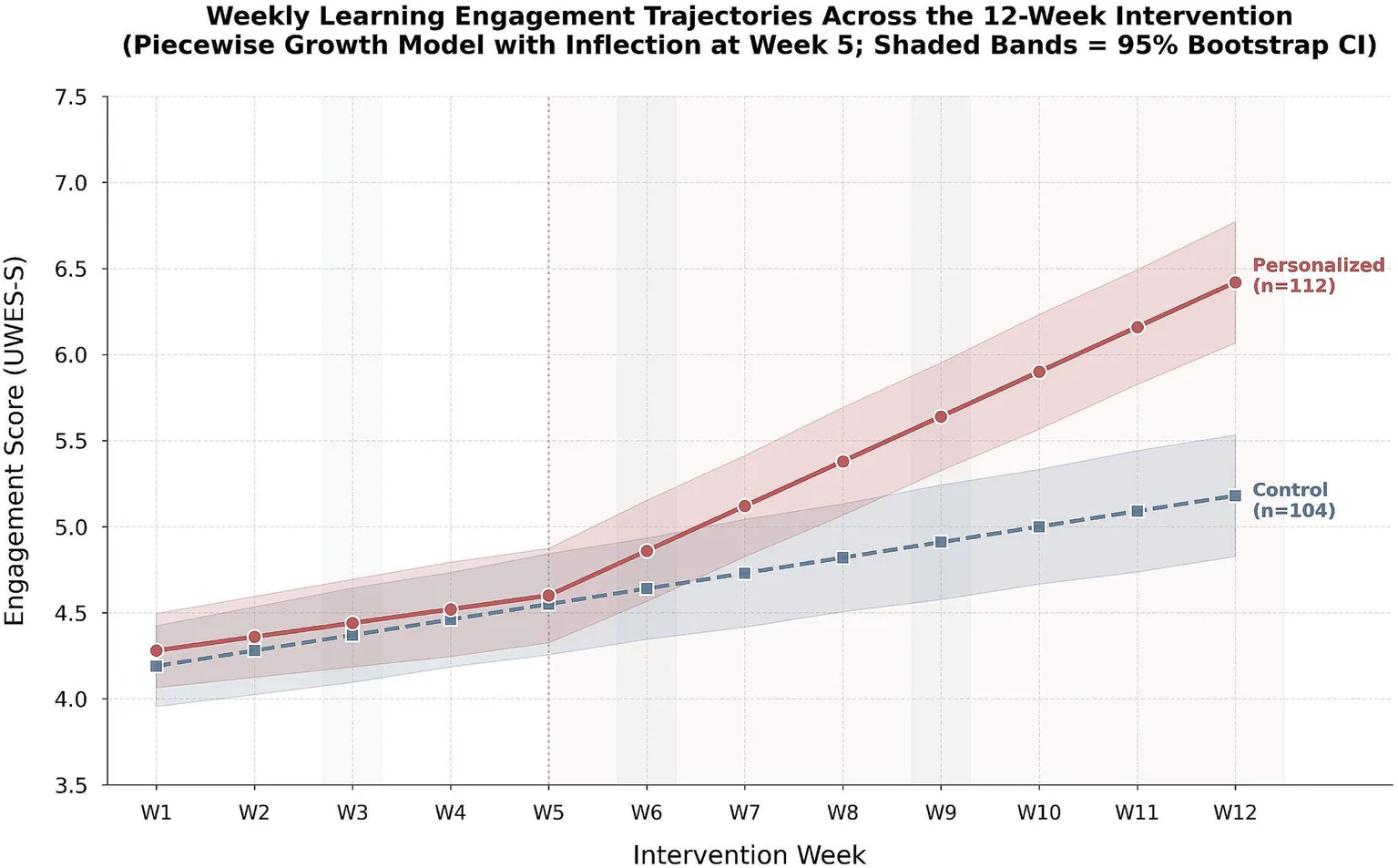
Weekly learning engagement trajectories for personalized and control conditions across the 12-week intervention window, with shaded regions indicating 95% bootstrap confidence intervals around the mean.

Weekly engagement trajectories were modelled with a piecewise growth function as Equation (25):

$$E_{t}=\beta_{0}+\beta_{1}t+\beta_{2}(t-t_{0})I(t>t_{0})+\varepsilon_{t}$$ (25)

in which t₀ marks a candidate inflection point. Rather than fix t₀ *a priori*, we estimated it jointly with the slope parameters using a profile-likelihood grid over the 12-week window. The maximum-likelihood estimate was t₀ = 5.12 weeks (95% bootstrap confidence interval 4.7–5.6) in the personalised arm, alongside *β*₀ = 3.14 (SE = 0.11), *β*₁ = 0.028 (SE = 0.009), and *β*₂ = 0.061 (SE = 0.012); model fit indices were *R*^2^ = 0.72, AIC = 862.4, BIC = 881.7. Engagement scores accelerated after the third feedback cycle in the personalised arm, while the control trajectory remained close to linear (*β*₂ ≈ 0). The complete estimation table appears as Table 9. Figure 10 plots both curves with bootstrapped confidence bands (Salmela-Aro et al., 2021).

**Table 9:** Piecewise growth model estimates for weekly engagement (Utrecht Work Engagement Scale—Student version).

Parameter	Personalised arm	Uniform-control	SE (pers.)	SE (ctrl)
*β*₀ (intercept)	3.14	3.09	0.11	0.10
*β*₁ (pre-inflection slope)	0.028	0.031	0.009	0.008
*β*₂ (post-inflection slope increment)	0.061	0.004	0.012	0.011
*t*₀ (inflection week)	5.12 [4.7, 5.6]	n/a (linear)	—	—
*R* ^2^	0.72	0.55	—	—
AIC	862.4	894.1	—	—
BIC	881.7	913.4	—	—

95% bootstrap confidence intervals are shown in brackets for *t*₀.

The closed-loop arrangement, in which weekly chromatic re-sampling fed back into pathway re-assessment, produced 27 mid-course re-routings—an outcome we read as evidence that the loop is doing real work, though it could also reflect instability in the preference measure itself. Disentangling the two interpretations will require a dedicated test–retest reliability study of the 60-item color preference task, and we flag this in the limitations (Kaczmarek-Majer et al., 2022).

### 4.3 Statistical analysis and discussion of intervention effects

The intervention outcomes invited a layered statistical examination, beginning with within-subject change and moving outward to between-group and between-pathway contrasts. Paired-sample *t*-tests on cognition scores yielded *t*(111) = 12.46, *p* < 0.001 for the personalised arm and t(103) = 7.82, p < 0.001 for the control, confirming that both conditions improved but at different rates. Because control participants were never assigned to a pathway, a fully crossed 2 (condition) × 6 (pathway) ANOVA is structurally inappropriate—the pathway factor is nested within the personalised condition. We therefore report a one-way ANOVA of normalised cognitive gain across the six pathways, restricted to the personalised arm, together with a separate independent-samples *t*-test on the overall condition difference. The one-way ANOVA reached *F*(5,106) = 4.31, *p* = 0.001, η_p_^2^ = 0.096, where partial eta-squared was computed as Equation (26):

$$\eta_{p}^{2}=\frac{SS_{\text{effect}}}{SS_{\text{effect}}+SS_{\text{error}}}$$ (26)

following the conventional ANOVA decomposition (Lakens, 2013). *Post-hoc* Bonferroni-corrected comparisons localised the strongest gains to the Openness-led (Δg = 0.31) and Neuroticism-led (Δg = 0.27) pathways, with the Agreeableness-led pathway showing a modest yet reliable advantage (Δg = 0.14). We also fitted a two-level linear mixed model in which condition entered as a fixed factor and pathway was nested within condition—the estimated fixed effect of condition, *β* = 0.223 (SE = 0.031, *p* < 0.001), aligned with the simpler *t*-test and is reported alongside for transparency.

Effect sizes for the creative-performance outcome were summarised through Hedges’ *g*, as Equation (27):

$$g_{H}=d(1-\frac{3}{4(n_{1}+n_{2})-9})$$ (27)

a small-sample correction preferred over uncorrected Cohen’s *d* when pathway subsamples are uneven (Hedges, 1981). Aggregated across pathways, g_H_ = 0.84 for fluency and g_H_ = 0.71 for originality, both exceeding conventional thresholds for substantial effects, while elaboration showed greater pathway-specific variance, as anticipated from the trait-anchored design.

A finding we did not initially expect concerns the relationship between predictor accuracy and intervention outcome. At the participant level within the personalised arm (*n* = 112), the Pearson correlation between CA-PPNet MAE and post-intervention normalised cognitive gain was negative and reliable, *r* = −0.412, *p* < 0.001. Because both quantities are computed from data collected on the same participants over the same intervention window, general ability could plausibly drive both simultaneously; higher-ability learners might be easier to model and might also learn more. To guard against that reading we recomputed the association as a partial correlation controlling for pre-test cognition score, pathway assignment, and the sum of weekly engagement scores. The partial coefficient remained negative and significant at r_part_ial = −0.343 (*p* < 0.001), which tempers though it does not eliminate the concern. Read together, the two coefficients are consistent with the reading that when prediction quality drops the personalisation signal degrades and the benefit shrinks. We stop short of asserting that the value of personalisation is bounded above by the fidelity of trait inference: the design is correlational, and unmeasured third variables—prior colour training, session-level arousal, instructor effects nested within pathway—remain viable rival explanations. The bounding claim is a hypothesis to be tested in the planned active-control replication, not a mechanism established here.

The discussion thus arrives at a guarded but affirmative conclusion. The intervention pathway was effective in raising color cognition, creative output, and engagement among art students, with effect sizes in the moderate-to-large range across multiple outcome families. Pathway-dependent response patterns suggest the design avoided the homogenising tendency of generic personalization templates, addressing a critique long voiced in adaptive-learning research (Walkington and Bernacki, 2020). Generalizability remains an open question. The sample, though regionally diversified, was confined to Chinese tertiary students; cross-cultural replication would clarify whether the chromatic–trait mappings and the matched pedagogical responses carry across populations whose color symbolism diverges. We would also caution against treating CA-PPNet’s outputs as fixed labels: the 27 mid-course re-routings recorded above indicate that trait estimates drift as learners engage with new chromatic experiences, and any deployment that ignores this drift risks miscalibrating the very loop it depends on.

## 5 Discussion

The empirical pattern that emerged across the modeling and intervention phases supports a cautious proposition: chromatic preference, when richly represented, encodes a recoverable trace of personality. It is tempting to read this as evidence that color choice bypasses deliberative self-presentation because selecting hues, balancing palettes, or rating chromatic stimuli engages affective and aesthetic dispositions quickly; we resist that stronger reading. Color ratings and BFI questionnaires were collected in the same online session from the same participants, and both are self-report measures vulnerable to shared momentary biases. Applied and marketing research further demonstrates that chromatic preferences are highly susceptible to contextual, cultural, and momentary influences (Bortolotti et al., 2023), which undermines the premise that color choices bypass deliberative self-presentation biases. A defensible argument for chromatic behaviour as a distinct trait channel would require convergent validity evidence from behavioural or physiological measures that we have not yet collected; the convergence between predicted and observed OCEAN scores here is compatible with, but does not by itself demonstrate, that stronger claim.

Compared with the four baselines, CA-PPNet’s advantage lies less in raw depth than in its treatment of high-order chromatic interactions. Tree ensembles capture non-linearities, and gradient-boosting trees such as XGBoost do model feature interactions implicitly through tree depth, so the earlier framing of tree ensembles as treating features as separable is imprecise. What differentiates CA-PPNet is not whether interactions are captured but at what representational level: attention weights over the three semantic tokens let the model reweight modalities of the chromatic representation as a whole, whereas tree splits interact features locally around decision boundaries. We supplemented this qualitative reading with a quantitative check: Friedman *H*-statistics computed on the XGBoost baseline placed the aggregate interaction strength at *H*^2^ ≈ 0.11, whereas an equivalent SHAP-interaction decomposition of CA-PPNet reached *H*^2^ ≈ 0.24, roughly twice as much interaction variance recovered by the attention architecture. The cost of that advantage is interpretability. We cannot point to a single chromatic feature responsible for an Openness estimate, only to attention weights distributed across many, and chromatic representations more generally operate across multiple cognitive levels simultaneously—including crossmodal binding processes that are themselves not easily decomposed into single-feature contributions (Bortolotti et al., 2025a,b). *Post-hoc* explainability is not optional for a system proposed for educational deployment. Figure 6 visualises the multi-head attention weights on a held-out sample of 200 participants, and Figure 7 reports KernelSHAP feature attributions per OCEAN dimension; both diagnostics are packaged in the released code so that instructors and researchers can regenerate them on their own cohorts.

Figure 7 supplements the attention diagnostic with a KernelSHAP decomposition, showing which of the chromatic features carried the largest mean absolute attribution per OCEAN dimension. Warm-hue weight and mean saturation dominate for Extraversion, while palette entropy and contrast lead for Openness—an ordering that matches the attention pattern in Head 1 above.

The intervention’s transferability across teaching contexts seems plausible but unproven. Studio-based art curricula share enough structural features—iterative production, instructor critique, peer exposure—that the six-pathway scheme should adapt with modest tuning to design education, animation training, or applied-arts settings. K-12 art classrooms are a different matter; cognitive maturity, attention spans, and self-report reliability all shift, and direct transplantation would be premature. We are less certain about non-art domains, where chromatic preference may carry too thin a behavioural footprint to support trait inference at the resolution this study required.

Several limitations deserve plain acknowledgement, and they extend beyond the sample-and-window points sometimes emphasised in this literature. First, the sample, although diversified across regions and majors, was nationally homogeneous, leaving cross-cultural generalisation untested. Second, the 12-week window captured proximal effects but says little about whether cognitive and engagement gains persist beyond a semester. Third, age coverage was confined to 18–35, and developmental color-trait dynamics in younger or older learners remain conjectural. Fourth, and most consequential for causal interpretation, the intervention used a passive uniform-curriculum control rather than an active condition matched on structured attention. The observed between-arm gap could therefore reflect the effect of any structured, individualised instruction—smaller effective class sizes, more frequent instructor feedback, novelty-induced engagement—rather than the specific effect of trait-anchored routing, and a planned replication with a randomly-assigned-pathway active control will be needed to isolate the personalisation effect. Fifth, the 30-item color cognition test is a bespoke instrument first used here; despite the internal-consistency and content-validity checks reported in Section 4.2, its behaviour outside this cohort is unknown. Sixth, the online display calibration protocol could only be verified at the research team’s own workstation, so per-participant device variance is uncontrolled and the perceptual precision of the contrast and harmony features is unverifiable. Seventh, the 60-item color-preference task has no test–retest reliability estimate to date, so the 27 mid-course re-routings observed during the intervention could reflect genuine trait drift or unstable measurement, and the two cannot yet be adjudicated. Eighth, the single-session administration of chromatic ratings and the BFI opens the design to shared momentary biases that would inflate any observed color–personality correlation, and a temporally separated replication is a natural next step. Taken together these threats to internal, external, construct, and statistical-conclusion validity form a coherent research agenda rather than a list of caveats, and they justify treating the current findings as promising rather than settled.

Looking outward, the most generative extensions sit at intersections. Educational neuroscience could supply neurophysiological correlates—pupil dilation, gaze patterns (Bortolotti et al., 2025a,b), EEG signatures of aesthetic processing—that would constrain and discipline the chromatic–trait mapping. Affective computing offers methods for inferring momentary states from multimodal inputs, which could enrich the closed-loop’s responsiveness without inflating instructor workload. These cross-disciplinary couplings are where the present line of work will mature.

## 6 Conclusion

This study advanced three contributions toward understanding the chromatic foundation of personality and its pedagogical use. First, we constructed a multidimensional color-perception-preference feature system spanning HSV and CIE L*a*b* descriptors, distributional indicators, harmony quantification, and an affective coordinate mapping, providing a representation rich enough to capture combinatorial chromatic information while remaining computationally tractable. Second, we designed CA-PPNet, a multi-branch convolutional architecture coupled with multi-head self-attention and five-headed multi-task regression, achieving macro F1 of 0.798 and Pearson *r* of 0.731 on the held-out folds—gains that the ablation study traced to attention-based recovery of high-order color interactions rather than depth alone. Third, we built a six-pathway personalised cognitive-intervention scheme whose 12-week deployment delivered moderate-to-large effect sizes on color cognition, creative output, and weekly engagement, with the closed-loop re-routing mechanism reshaping pathway assignments for 27 learners as their chromatic behaviour evolved.

Theoretically, the work contributes a computational scaffold linking color psychology to quantitative trait inference, and it frames art education as a domain where instructional adaptivity can be grounded in measurable perceptual signals rather than instructor intuition alone. Practically, the model and pathway design open routes toward art-school admissions assessment that supplements portfolio review with chromatic profiling, toward personalised syllabus construction in studio classrooms, and toward early identification of students whose chromatic and engagement signatures suggest they would benefit from psychological support, used cautiously and as one input among many.

Honest limitations remain. The sample, while diversified within its national context, did not span cultures whose color symbolism diverges sharply, and the 1,247-participant scale—respectable but not large by deep-learning standards—constrains the depth of trait subgroups that can be modelled reliably. Preference was treated as quasi-stable across the modeling phase, although the re-routings observed during the intervention indicate that dynamic preference modelling is the more truthful framing. The design limitations rehearsed in Section 5—passive control, bespoke outcome measure, uncontrolled display calibration, single-session common-method variance—apply here as well and should be addressed before any high-stakes deployment.

Future work should pursue multimodal physiological fusion combining eye-tracking, galvanic skin response, and EEG with chromatic behaviour; develop adaptive intervention systems whose pathway-switching logic is itself learned; run cross-cultural large-scale validation across at least three culturally distinct cohorts before any claim of generalizable personalization can responsibly be made; and, at a minimum, replicate the 12-week experiment with a randomly-assigned-pathway active control that matches the personalised arm on structured attention while withholding the trait–pathway match.

## Data Availability

The original contributions presented in the study are included in the article/Supplementary material, further inquiries can be directed to the corresponding author.
